# Comparative Composition, Diversity and Trophic Ecology of Sediment Macrofauna at Vents, Seeps and Organic Falls

**DOI:** 10.1371/journal.pone.0033515

**Published:** 2012-04-04

**Authors:** Angelo F. Bernardino, Lisa A. Levin, Andrew R. Thurber, Craig R. Smith

**Affiliations:** 1 Departamento de Oceanografia e Ecologia, Universidade Federal do Espírito Santo, Goiabeiras, Vitória, Espírito Santo, Brazil; 2 Center for Marine Biodiversity and Conservation; Integrative Oceanography Division, Scripps Institution of Oceanography, La Jolla, California, United States of America; 3 College of Earth, Ocean, and Atmospheric Sciences, Oregon State University, Corvallis, Oregon, United States of America; 4 Department of Oceanography, School of Ocean and Earth Science and Technology, University of Hawaii, Honolulu, Hawaii, United States of America; UC Merced, School of Natural Sciences, United States of America

## Abstract

Sediments associated with hydrothermal venting, methane seepage and large organic falls such as whale, wood and plant detritus create deep-sea networks of soft-sediment habitats fueled, at least in part, by the oxidation of reduced chemicals. Biological studies at deep-sea vents, seeps and organic falls have looked at macrofaunal taxa, but there has yet to be a systematic comparison of the community-level attributes of sediment macrobenthos in various reducing ecosystems. Here we review key similarities and differences in the sediment-dwelling assemblages of each system with the goals of (1) generating a predictive framework for the exploration and study of newly identified reducing habitats, and (2) identifying taxa and communities that overlap across ecosystems. We show that deep-sea seep, vent and organic-fall sediments are highly heterogeneous. They sustain different geochemical and microbial processes that are reflected in a complex mosaic of habitats inhabited by a mixture of specialist (heterotrophic and symbiont-associated) and background fauna. Community-level comparisons reveal that vent, seep and organic-fall macrofauna are very distinct in terms of composition at the family level, although they share many dominant taxa among these highly sulphidic habitats. Stress gradients are good predictors of macrofaunal diversity at some sites, but habitat heterogeneity and facilitation often modify community structure. The biogeochemical differences across ecosystems and within habitats result in wide differences in organic utilization (i.e., food sources) and in the prevalence of chemosynthesis-derived nutrition. In the Pacific, vents, seeps and organic-falls exhibit distinct macrofaunal assemblages at broad-scales contributing to ß diversity. This has important implications for the conservation of reducing ecosystems, which face growing threats from human activities.

## Introduction

Reduced (or ‘reducing’) sediments occur where anaerobic metabolism or geochemical processes provide a flux of reduced inorganic chemicals (e.g., sulfide, methane) that fuel chemoautotrophic production. Such sediments are widespread in wetlands, estuaries and organic-rich shelves, and on continental slopes beneath regions of high primary productivity. They are also found at sites of hydrothermal venting, methane seepage and large organic falls such as whale, wood and algal detritus. Although a variety of chemicals co-occur in these soft sediment ecosystems, H_2_S is typically elevated and plays a key role in structuring faunal communities. Sulfide is toxic to most metazoan taxa [1], [2], although some sediment-dwelling taxa have adapted to conditions of low oxygen and appear capable of tolerating the presence of sulfide. Due to high local production, metazoans in reducing sediments in the deep sea are often released from the extreme food limitation prevalent in the background community (e.g. [3]). Instead, chemical toxicity may drive infaunal community structure. In this meta-analysis we ask which taxa are common across these soft-sediment reducing ecosystems in the deep sea, and infer the role of oxygen and sulfide in structuring these food-rich “oases”.

Methane seeps, sedimented hydrothermal vents and organic falls are patchily distributed; they occur most frequently near ocean margins from intertidal to hadal depths [4], [5], [6], [7], [8]. Whale falls are most likely to be common along whale migration routes, kelp falls adjacent to coastal kelp beds, and wood falls, though very widespread, are likely to be most common along forested margins and near the mouths of rivers draining forested ecosystems. Hydrothermal vents occur along tectonic plate boundaries including both spreading centers and back arc basins, but only subsets of these habitats have soft sediment overlying the recently extruded basalts or precipitated sulfides. Methane seeps are common along continental margins in areas of high primary productivity and tectonic activity, where crustal deformation and compaction drive emissions of methane rich fluid [9]. Together, these ecosystems create a network, extending along margins and across ocean basins, of soft-sediment habitats fueled, at least in part, by the oxidation of reduced chemicals.

Biological studies at vents, seeps and organic falls initially focused on hard substrates and megafaunal taxa, especially those with chemoautotrophic symbionts [10], [11], [12]. The study of sediment biota at vents and seeps in particular, and to a lesser extent kelp, wood and whale falls, developed more slowly [13], [14], [15], [16], [17], [18], [19], [20]. Although scientists working in multiple reducing systems have studied similarities between symbiont-bearing and megafaunal communities (e.g. [21], [22], there has yet to be a systematic comparison of the community-level attributes of sediment macrobenthos across deep-sea reducing ecosystems.

Here we compare the community structure, function and dynamics of macrofaunal invertebrates (>300 µm) inhabiting sediments at methane seeps, hydrothermal vents, and surrounding whale, wood and kelp falls at water depths >200 m. Vent and seep biota below 200 m typically exhibit much greater systematic specialization and reliance on chemoautotrophy than those from shelf depths [15], [23]. While there is a growing literature on the metazoan meiofauna and protozoa at seeps and whale falls, we limit our synthesis to the macrobenthos for which there are a large number of samples analyzed with relatively standard approaches. Quantitative comparisons are limited to the Pacific Ocean, where parallel data sets were available across a range of reducing ecosystems. Our review evaluates key similarities and differences in the sediment-dwelling assemblages of each system with the goals of (1) generating a predictive framework for the exploration and study of newly identified reducing habitats, and (2) identifying taxa and communities that overlap across ecosystems.

Gradients in reducing activity are generated through distance from organic or vent/seep sources, and from temporal changes in seepage, venting, or organic decay processes. In most systems, the biotic response to flow or seepage through sediments generates recognizable biogenic habitats such as bacterial mats, pogonophoran fields, vesicomyid and clam beds, or successional stages linked to sulfide availability [24]. Previous within-habitat studies have shown that the sediment faunas within these microhabitats can be distinct [25] although some may be a subset of others [20].

Given that sediment microbiological and geochemical properties are likely to be drivers of infaunal assemblage structure, we first ask: What are the commonalities and differences in biogeochemical conditions of the various vent/seep/organic-fall soft-sediment habitats and successional stages? *We hypothesize that similarities in sulfide pore-water distributions, methane availability and temperature will promote comparable macrobenthic assemblages and nutritional pathways*. We then assemble and synthesize macrobenthos data from sediment cores taken in different reducing ecosystems. We ask whether there are aspects of community structure, including patterns of abundance, taxonomic composition, diversity or lifestyles, shared across macroinfaunal assemblages of vents, seeps and organic falls. *We hypothesize that all systems at high sulfide concentrations will exhibit enhanced density, reduced diversity, and shared families and genera of symbiont-bearing and heterotrophic taxa*. For those systems for which stable isotope data have been collected, we assess trophic pathways, including the relative contributions of chemoautotrophic and photosynthetic production, the contributions of methane, and the importance of sulfide oxidation in food chains. Finally, we evaluate the implications of ecological similarities and differences for levels of endemicity of the fauna associated within these ecosystems.

## Methods

### Data sets

#### Seeps

With the recent discovery of a methane seep in the Southern Ocean [26], methane seeps are now known in all oceans [10]. However, comparable macro-infaunal data are limited to the northeastern and southwestern Pacific Ocean, the Gulf of Mexico, and the eastern Atlantic (Table 1). The sites examined here range from 400 m to 4480 m water depths, with the greatest number from along the Eastern Pacific margin stretching from Costa Rica to the Aleutian Islands.

**Table 1:** Global chemosynthetic ecosystems and study sites where sediment macrofaunal data were available and analyzed in this study.

Reducing ecosystem sediments	Region	Location	Water depth (m)	Habitats	Data source
Middle Valley (HV)	E. Pacific	Juan de Fuca	2406–2411	mat, clam bed, hot mud, inactive	[27]
Papua New Guinea (HV)	W. Pacific	Manus Basin	1430–1634	active and inactive sediments	[27]
Gulf of Alaska (MS)	NE Pacific	Kodiak Seep	4327–4480	frenulate field, clam bed, non seep	[80]
Aleutians (MS)	N. Pacific	Unimak Seep	4500	frenulate field, clam bed, non seep	[80]
Oregon Margin (MS)	E. Pacific	Hydrate Ridge	770	mat, clam bed, near seep, non seep	[20]
California Margin (MS)	E. Pacific	Eel R. Seep	500-252	mat, clam bed, near seep, non seep	[20], [61], [109]
So. California Borderland (MS)	E. Pacific	San Clemente	1800	frenulate field	[90]
Costa Rica (MS)	E. Pacific	Quepos, Mound 12 and 11, Jaco	400, 990, 1020, 744–1795	mat, clam bed (little)	Levin and Mendoza, unpublished.
New Zealand (MS)	W. Pacific	N. Island Seeps	662–1201	ampharetid bed, frenulate field	[63]
Gulf of Mexico (MS)	Gulf of Mexico	Florida Escarpment	3234–3290	mat, frenulate field, clam bed, non seep	[80]
So. California Borderland (WF)	E. Pacific	Santa Cruz Basin	1670	6 wk, 18 mo, 4.5 y, 5.8 y, 6.8 y	[29]
So. California Borderland (KF)	E. Pacific (Kp)	Santa Cruz Basin	1670	<1 m, 3 mo., 6 mo.	[17]
So. California Borderland (WO)	E. Pacific (Wd)	Santa Cruz Basin	1670	<1 m, 6 mo, 22 mo, 3 y, 5.5 y	[17]

HV- Hydrothermal vents; MS – Methane Seeps; WF- Whale-fall; KF- Kelp-fall; WO- Wood-fall.

#### Vents

While the majority of hydrothermal vent habitats are primarily hard substrate, a number of known vents sustain hydrothermal fluids efflux through seafloor sediments. The infaunal macrobenthos from vent systems in this study include sedimented vents from Guaymas Basin (1800–2000 m), Escanaba Trough (3250 m), Middle Valley (2400 m), Galapagos Mounds (2700 m), and Manus Basin (1430–1630 m; Table 1). The most detailed quantitative analyses of macrofauna have been conducted in Middle Valley, NE Pacific [27] and at the Solwara and South Su mining exploration sites within Manus Basin, SW Pacific.

#### Organic falls

The most complete successional study of infaunal macrobenthos at a whale fall was conducted in the NE Pacific on an implanted 30-ton gray whale (*Eschrichtius robustus*) studied over 7 years at 1670 m depth in Santa Cruz Basin [16], [28], [29] (Table 1). Sediment samples were collected at 0 to 100 m distances from the whale carcass, allowing comparisons of community structure at different levels of organic enrichment and sulfide concentration [30], [31], [32]. Successional studies of most other whale carcasses have used imaging or bone collections to focus on megafauna and bone epifauna so these were not included in this study [33], [34], [35]. The effects of organic enrichment from wood and kelp falls on the sediment macrofauna were quantitatively studied in the NE Pacific, where wood and kelp falls (100 and 200 kg each, respectively) were deployed at 1670 m depth and revisited after 0.25 to 5.5 y [17]. Sediment samples were taken at 0, 0.5, 1,2 and ∼100 m distances, providing the first robust understanding of infaunal dynamics at these organic-fall types in the deep NE Pacific (Table 1).

### Data analysis

**Figure 1:**
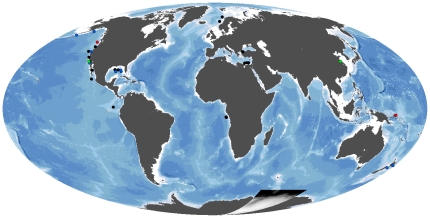
Global distribution of known chemosynthetic ecosystems. Colored dots represent quantitative faunal studies at hydrothermal vents (red), cold seeps (blue), and organic falls (green). Black dots indicate chemosynthetic sites used for comparisons only.

Macroinfaunal abundance and composition were statistically compared between similar habitats across vents, seeps and organic-falls (Figure 1, Table 1). Community analyses were mainly performed on datasets that had species counts and replicated samples from single habitats, which limited statistical analysis to the examined data sets. Although most sites where species abundance matrices were available were included in the data analysis (Table 1); we added comparisons to the literature available from many other sites and regions (Table S1). Abundance data were normalized to 1 m^2^, and each core sample treated as a replicate from a single habitat (see below). Due to different sampling efforts, the number of replicates within each habitat per site varied (N = 2 to 20), and therefore samples from ecologically comparable depth ranges and succession stages (at organic falls) within each habitat were combined in order to facilitate statistical analysis. For organic-fall habitats, we included only samples adjacent to experiments (0 m), where reducing conditions are likely to be most intense at any particular time point (e.g., [31]). Although sediments at greater distances (up to 10 meters) exhibit distinct macrofaunal communities compared to background deep-sea sediments, they likely represent different reducing conditions and habitat types. For the whale fall, data are included from time points of 4.5, 5.8 and 6.8 yr; for wood falls from 3 and 5.5 yr, and for kelp-falls from 0.25 yr after emplacement [17], [32]. Statistical analyses of macrofaunal density were conducted with ANOVA after tests for homogeneity of variances. For significant ANOVA results, *post-hoc* tests were used to examine difference in means using the statistical package BioEstat©. Macrofaunal composition was generally compared at the family or higher levels, yielding up to 73 distinct taxa within at least one sample. Species diversity was evaluated for pooled replicate cores at each habitat and site sampled (n = 1–4) due to the low density of metazoans. Hulbert's (1971) [36] modification of Sanders rarefaction (ES_n_) was used to compare species diversity between treatments.

Non-metric multi dimensional scaling (MDS) and Cluster techniques were used to compare community structure across habitats (Microbial mats, Clam beds, Hydrothermal muds, Frenulate fields, Organic-rich sediments) and chemosynthetic systems (Vents, Seeps and Organic falls; Table 1). Comparisons were made based on Bray-Curtis similarities calculated from fourth-root transformed, family abundance data from standardized quantitative samples (PRIMER; [37]). Analysis of similarity tests (ANOSIM routine) was used to determine significant differences among groups identified by cluster and MDS techniques. Dissimilarity values in fourth-root transformed, standardized quantitative data were obtained from SIMPER analysis [37]. Based on multivariate dispersions from resemblance matrixes at all sites, we tested the null hypothesis of no differences in within-group dispersion among groups using the PERMDISP routine [38]. This routine also allowed testing for differences in beta diversity among sites (Vents, Seeps and Organic-falls) and habitats, based on Bray-Curtis resemblance on presence/absence data (Jaccard's dissimilarity index) from all sites (PRIMER; [39]).

## Results

### Biogeochemical processes

#### Vents

Hydrothermal venting through sediments is associated with elevated sediment temperatures and sulfide concentrations, and the occasional presence of microbial mats or vesicomyid clams. Environmental and biogeochemical processes in deep-sea hydrothermal vent systems differ significantly from background sediments [40], [41]. The pronounced temperature differences among vent sites typically occur due to different sizes of venting areas and variable diffusive fluid flow within habitats, such as active sites near venting chimneys, inactive vent sediments or microbial mats [23], [27], [42], [43]. Deep-water hydrothermal fluids can exceed temperatures of 400°C [23]. Although the vent benthic biota is usually found in temperatures between 10 and 25°C [44], temperatures up to 94°C can occur in the top 5 cm in the sediment column in vent habitats [27]. Concentration of inorganic chemicals in deep-water vent fluids vary significantly within venting regions (reviewed by [40]), but at active vent sediments there are usually high concentrations of CH_4_, H_2_S, H_2_ and metals [23]. End member vent fluids are enriched in sulfide (typically 1.5–8 mM) formed by thermal sulfate reduction and basalt leaching above 250°C [45], while methane concentrations are typically sub-millimolar in concentration [46].

Sedimented hydrothermal areas can be regarded as geochemically similar to methane seeps but with elevated temperatures in pore fluids and sediments. Compared to basalt-hosted hydrothermal vents, they are more enriched in methane (up to 2 orders of magnitude) [47] and in hydrogen sulfide but generally are less rich in reduced metals. Sediment-covered vent fields are significantly lower in temperature than bare-rock vents. Sediments appear to facilitate subsurface cooling and reequilibration of high-temperature fluids before venting occurs at the sediment-water interface. In contrast to basalt-hosted hydrothermal vents, fluids in sedimented areas are enriched in methane and hydrogen due to thermogenic decomposition of sedimentary organic matter [48]. High organic matter input also enhances microbial sulfate-reduction rates, leading to enhanced sulfide concentrations in sediments [49], [50], [51]. Chemoautotrophic bacteria frequently colonize hydrothermal-vent sediments and are considered an important food source for macrofaunal organisms at some vent sites [27], [40]. Sulphur-oxidizing filamentous bacteria dominate at many vent sites, but the occurrence of methanotrophic bacteria is also common in sedimented Atlantic and Pacific vents [23].

#### Seeps

Methane seep habitats consist of a continuum from background to highly sulphidic sediments associated with methane emission. As methane is released from deep-subsurface reservoirs along subsurface deformations and faults, it can be initially oxidized anaerobically by a syntrophic consortium of bacteria and archaea. This process, the anaerobic oxidation of methane (AOM), commonly uses sulfate as the electron acceptor (but see [52]), to produce hydrogen sulfide as methane is both respired and its carbon used to build the microbial consortium biomass [53], [54], [55]. These consortia are composed of methanotrophs (Euryarchaea) and sulfate reducing bacteria. As both methane and sulfide, the latter formed from AOM, reach oxygenated sediments or the overlying water column, aerobic sulfide oxidation and methanotrophy (methane oxidation) provide two additional pathways of carbon fixation, both of which are carried out by bacteria (including α-, δ- and γ- proteobacteria; [56], [57]). As in all of the other reducing habitats, the sulfide-oxidizing bacteria can be large enough to be visible to the naked eye, sometimes creating microbial mats. Mat constituents exhibit mixotrophy, combining chemoautotrophic production with heterotrophy [58].

In addition to microbial mats, a variety of other habitats occur at methane seeps, which are identified by their dominant megafauna and result from different underlying biogeochemistry. Microbial mats commonly sustain high methane emission rates and the greatest concentrations of sulfide (reaching >20 mM concentration within the surface sediments). Clam beds (inhabited by chemosynthetic, symbiont-bearing vesicomyid and solemyid clams) and fields of frenulates (siboglinid polychaetes previously referred to as pogonophora) are characterized by lower sulfide concentrations near the sediment surface [15], [19], [59]. Clam beds also have lower and/or oscillating fluid flow compared to bacterial mats [60], [61]. The clams themselves bio-irrigate the sediment, extending oxygen penetration to 3 to 6 cm below the sediment surface [62]; in microbial mats oxygen penetration is <1 cm. In certain habitats, ampharetid polychaetes occur in great densities (>35,000 individuals m^−2^; [63]). The ampharetid tubes may facilitate upward transport of methane as this habitat exhibits the highest methane emission rate known from non-bubbling sites (>200 mmol methane m^−2^ day; [64], [65]. These four seep habitats reflect geochemical and trophic heterogeneity on <1 m scales.

**Table 2:** Sediment organic content and maximum sulfide for kelp-, wood- and whale-falls.

Habitat	Fall size	Sed TOC %	Sed TON % (1 SE)	C/N	Porewater sulfide	Radius/Time of influence	Ref.
Kelp falls	100 Kg	7.6–7.7	0.8 (0.02)	11.9	1 mmol.L-1	0 m/3 mo	[17]; C.R. Smith unpublished
Wood falls	200 Kg	26.8–29.2	0.4 (0.02)	81.6	n.a.	0 m/3 yrs	[17]; C.R. Smith unpublished
Whale fall	30 ton	7.2–14.2	0.8	14.4	1–8 mM	0 m/4.5 yrs	[31]
	n.a.	1.4–3.4	0.2–0.5	∼6.8	up to 29 µM	0 m/0.7–4.3 yrs	[74]

n.a. not available.

The different biogeochemistry that underlies each of the seep habitats leads to distinct trophic signatures in the fauna. Methane can be formed either through geologic processes or through AOM; this latter processes may be the dominant source of methane for seep systems [9]) and provides a key mechanism to track the role of methane-fueled production. Biogenic methanogenesis, or methane formed by AOM, imparts a highly skewed ratio of C^12^ to C^13^, favoring the lighter isotope. This unique isotopic ratio (C^12^/C^13^) provides a mechanism to identify animals that consume this type of production, as an animal's carbon isotopic signature is derived from their diet. While AOM mediated methanogenesis results in the most negative isotopic signature (referred to as δ^13^C when the ratio of ^13^C to ^12^C is compared to a standard), the other sources of fixed carbon, including sulfide oxidation, sulfate reduction, aerobic methane oxidation, as well as photosynthetic production from the overlying waters, all impart a characteristic, although often overlapping, δ^13^C signature [66]. Carbon is fixed at seeps through a variety of pathways including the rTCA cycle and the Calvin-Benson-Bessham cycle [67]. Thus, stable isotope studies have provided insights into the use of the two methane-based chemoautrophic production pathways (e.g. [63], [68], [69] and unique biomarkers present in aerobic methanotrophic bacteria have clearly shown the use of methanotrophic production by seep meiofauna and macrofauna [70], [71]. The role of anaerobic methanotrophy remains enigmatic, although isotopic evidence does suggest that archaeal biomass associated with AOM is consumed by infauna living in microbial-mat and carbonate habitats [68], [72].

#### Organic falls

The nature and mass of individual organic falls at the deep-sea floor may have major effects on decomposition rates, and ultimately influence food availability for benthic microbes and invertebrates at the fall site [30]. Carcasses of dead whales can deliver over 30 tons (per whale) of fresh organic material to the deep-sea floor [73]. As a result of rapid dispersal of flesh from the carcass by scavengers, and the intrusion of bones and soft tissue into sediments at deposition, the sediments around whale carcasses become massively enriched in organic material (Table 2, [16], [31]). Anaerobic degradation of organic matter and subsequent production of sulfides and methane within the sediments around the whale fall, support rapid (<18 months) development of chemoautotrophic assemblages in whale-fall sediments [16], [31], [32]. In organic-rich sediments near a whale fall, sulfate-reduction rates can increase by 1 to 3 orders of magnitude compared to background sediments, reaching 300 to 700 mmol m^−2^ d^−1^ [31] and yielding sulfide concentrations up to 20 mM [16], [31]. Methane concentrations also increase dramatically near whale falls, indicating that methanogenesis is an important degradation pathway within the whale-fall influenced sediments [31], [74].

Wood is composed of high concentrations of relatively refractory organic materials, including cellulose and lignin [75]. In the deep sea, initial decay of this refractory material is mediated by wood-boring *Xylophaga* bivalves and decomposition is mediated by fungae and bacteria [76], [77], [78]. Microbial succession and state of wood decomposition within wood parcels may be environmentally linked to immersion period, oxygen concentrations and wood type [76], which alter the physical and biochemical properties of the substrate. The release of particulate organic matter and *Xylophaga* fecal material to sediments nearby wood parcels leads to organic enrichment, anaerobic microbial metabolism, and modest enhancement of pore-water sulfide concentrations in nearby sediments (up to 0.015 mM, Table 2) [49].

Kelp parcels contain much more labile organic material than wood, and thus are scavenged by invertebrates and decomposed by microbes at much higher rates than wood falls of similar mass [17], [79]. As a result, particulate organic material is rapidly released to underlying sediments and creates organic-rich patches and increases pore-water sulfides concentrations within 3 months (up to 1.5 mM HS; [30]), allowing the development of microbial mats [17]. The dynamics of organic-matter release and microbial sulfide production, and their influence on macrofaunal succession in sediments, will be discussed below.

### Invertebrate community structure: Macrofaunal abundance and composition

#### Vents

**Figure 2:**
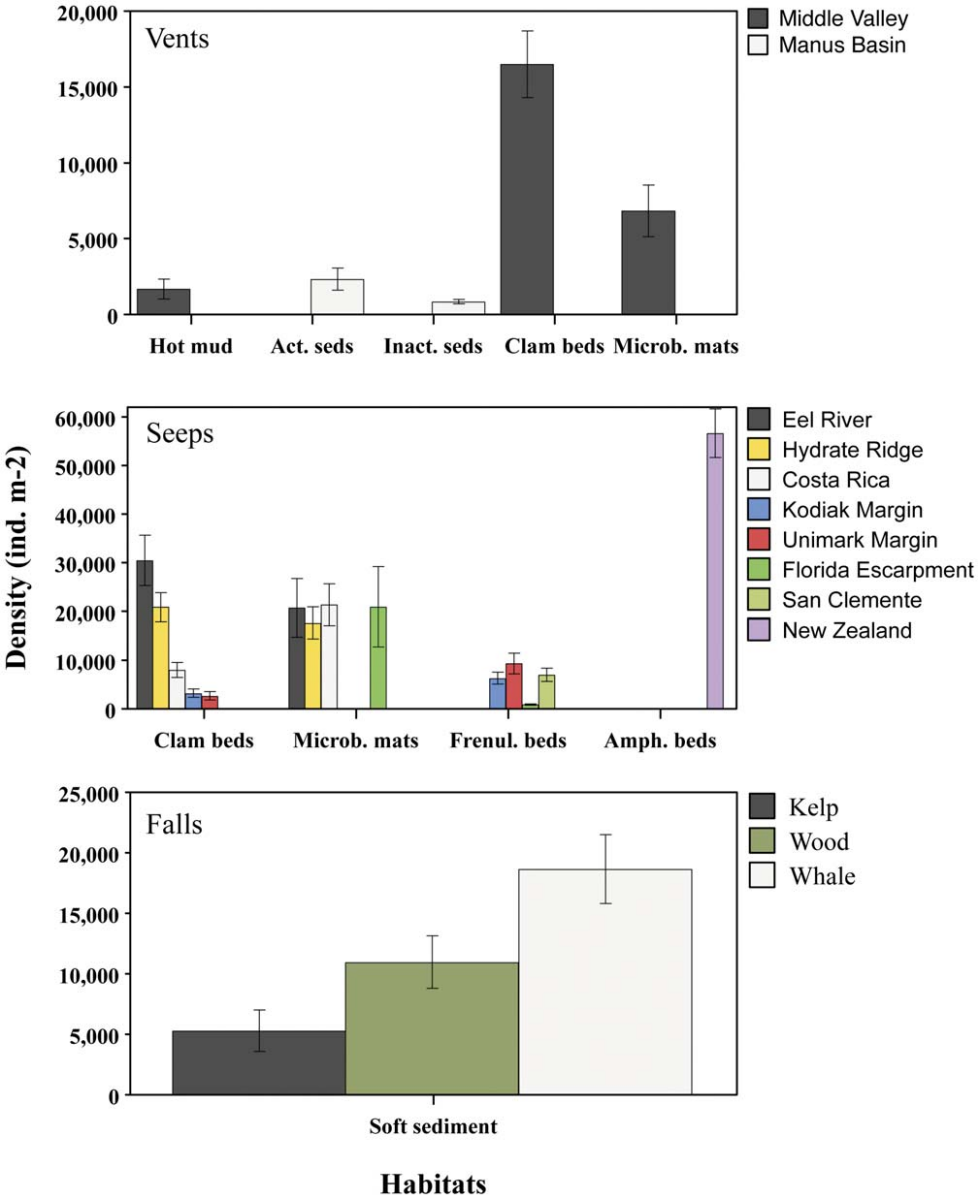
Macrofaunal density per habitat across Vents (upper panel), Seeps (middle panel), and Organic-Fall (lower panel) ecosystems. Average values (±1 SE).

Based on a limited number of study sites, there is no consistent enhancement of macrofaunal density or biomass at hydrothermally active sites relative to nearby inactive sites (Table S1). Faunal density responses appear to be a function of stress level. For example, at Middle Valley (2410 m), extremely hot sediments (e.g., 94°C at 5 cm into the sediment column) support very few macrofauna, whereas moderately warm sediments inhabited by vesicomyid clams may have elevated macrofaunal densities (16,500 ind m^−2^) relative to those in microbial mats (6,840 ind m^−2^), hot sediment (1,690 ind m^−2^), and control sediments (2,218 ind m^−2^; F = 29.9, P<0,001; Figure 2). Biomass differences among macrofauna in Middle Valley habitats are less dramatic (and not statistically different) but exhibit similar ranking to density [27]. In Manus Basin, macrofaunal densities were low at two inactive sites and one active site (<1,000 ind m^−2^), but significantly elevated at another active site (South Su - 3,494 ind m^−2^), due to the presence of relatively large spionid polychaetes and nuculanoid bivalves, which elevated biomass was 100-fold relative to a nearby inactive site.

**Figure 3:**
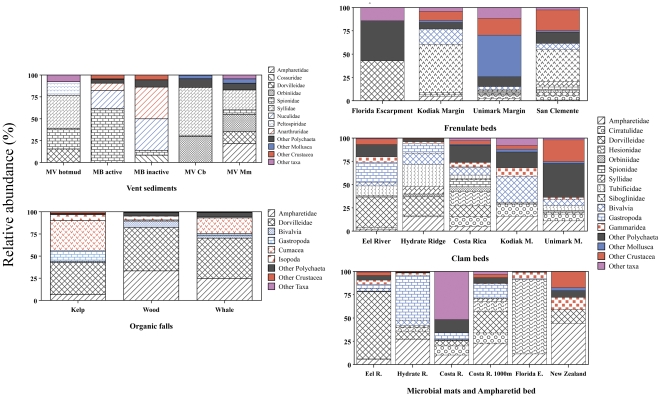
Macrofaunal composition within habitats in Vent, Seep and Organic-Fall ecosystems. Values are relative abundance (%) of all samples within each habitat/site. Color-code: Polychaetes (patterns in black); Mollusks (in blue); Crustaceans (in red) and Other taxa (purple). Ampharetid beds represented only in New Zealand.

Hydrothermal sediments with elevated densities are characterized by high dominance and an absence of large numbers of rare species (Table S1, Figure 3). In some instances, spionid polychaetes (genus *Prionospio* (*Minuspio*)) dominate (20–60% of abundance at South Su in Manus Basin and Middle Valley hot mud; Figure 3). Syllid polychaetes (*Sphaerosyllis* sp.) are also abundant in Middle Valley hot-mud sediments, as well as in clam beds and microbial mats. Nuculanid bivalves (*Nuculana* spp.) are a widespread group common in Manus Basin active and inactive sediments, together with tanaid and isopod crustaceans. Orbiniid, ampharetid, dorvilleid and hesionid polychaetes are also well represented in hydrothermal sediments of the E. Pacific (Table S1; Figure 3).

#### Seeps

Seep habitats commonly have increased macrofaunal density compared to background sediments (e.g., [80]), yet there are exceptions to this rule, particularly at upper bathyal depths (Figure 2). Infaunal densities for seep macrofauna range from 2,400 (San Clemente methane seep) to 81,400 ind m^−2^ (Ampharetid beds, New Zealand) with a mean of 20,200±860 ind m^−2^. The macrofauna of these habitats are far more dense than background sites, which ranged from 260 ind m^−2^ (Gulf of Mexico, 3300 m) to 19,760 ind m^−2^ (Eel River, 500 m), with a mean of 8,180±870 ind m^−2^. In the four geographic locations with microbial mats and clam beds, two had microbial-mat macrofaunal densities <0.8 times that of clam beds, while one (Costa Rica) having over 2.0 times the density in microbial mats compared to clam beds. Frenulate fields in all cases had increased macrofaunal density compared to reference sites. Ampharetid beds had the highest density of any habitat, with a mean density on the New Zealand margin that was over 13 times greater than an off-seep reference station.

Seep infauna includes a subset of background taxa apparently tolerant of high sulfide or with behaviors to avoid its toxicity. Within the Polychaeta, the Dorvilleidae are an abundant component of seep habitats, attaining the highest dominance at microbial mat habitats at Eel River seeps, and in frenulate fields in the Gulf of Mexico and on the Norwegian margin (Table S1, Figure 3). Their radiation and tolerance to sulfide is reflected in the presence of 30 species of dorvilleids at bathyal seeps off CA and OR ([15]; unpubl). Ampharetid and hesionid polychaetes also appear to be well suited to a wide variety of seep habitats and inhabit the sediment-water interface, likely as a way to minimize sulfide stress. The ampharetids, while present in most seep habitats, were dominant at Oregon, New Zealand, and Costa Rica margin seeps, comprising 24±4%, 46±6% and 14±3%, of the fauna at these locations, respectively (Figure 3). Ampharetids were also common in the Gulf of Guinea and at the Håkon Mosby volcano [25]. However they were not as abundant in areas with lower sulfide concentrations, such as frenulate fields; they were absent from half the samples collected.

While both the dorvilleids and the ampharetids were widespread among the seep habitats, certain groups were dominant in just one or a few locations. Microbial mats at seeps on the Oregon, Florida, and Costa Rica margins had uniquely high relative densities of gastropods (63%), hesionids (79%) and hydroids (20), respectively. There was an increase in diversity in less sulphidic habitats, such as clam beds and some frenulate fields, with cirratulid, spionid, syllid, and tubificid polychaetes as well as gastropods, amphipods, and cumaceans, present at many of these locations (Figure 3, Table S1). Thus, seep sediments host a broad range of families including those adapted to highly sulphidic seep habitats (e.g., dorvillieds), groups dominant in only specific conditions and at particular seep localities (i.e. hesionids), or taxa that sustain enhanced abundance associated with higher productivity around seeps (e.g, ampharetids).

Biomass is frequently higher in seep sediments than non-reducing habitats. In the Nile delta, microbial mat infaunal biomass was 3750 times that of referenced sites [69]. The highest biomass of heterotrophic fauna was found in New Zealand ampharetid beds with a maximum of 278 g m^−2^ [71]. At Hydrate Ridge, Oregon, the maximum biomass, including symbiont bearing fauna, was present in vesicomyid clam beds, 161±50 g m^−2^, biomass in beds of the solemyid clam *Acharax* was also high (143±67 g m^−2^). At Hydrate Ridge, biomass was moderate in microbial mats (46±23 g m^−2^), and lowest at reference sites (10±5 g m^−2^; [19]). On Hydrate Ridge, sites with the highest sulfide concentrations had modest biomass by seep standard, yet enhanced macrofaunal abundance.

#### Organic falls

The macrofaunal abundance in organic-rich sediments around whale, wood and kelp falls was consistently higher than in background sediments. At a 30-ton whale carcass in Santa Cruz Basin, California, macrofaunal densities adjacent to the whale (0 m) reached 41,596 ind m^−2^ at 6.8 yr, with a mean of 18,653 ind m^−2^ in the 4.5 to 6.8 y time frame (Table S1, Figure 2). Meter-scale patches of organic-rich sediments produced high heterogeneity in infaunal abundances and porewater sulfide concentrations around the whale carcass; with the highest macrofaunal densities up to 53-fold greater than background levels (780 ind m^−2^). Similar peaks in macrofaunal abundances (21,000–45,000 ind m^−2^) were observed in sediments nearby whale falls in San Diego Trough and Monterey Bay at 0.33 to 2 yr (Table S1). At kelp falls, macrofaunal densities are enhanced (5,286±997 ind m^−2^) over spatial scales of ≤1 m for at least 0.5 y. Five-fold increases relative to background sediments were observed (up to 8,320 ind/m^2^), especially within organic-rich, but relatively sulfide-poor, sediments. Macrofaunal densities in sediments around wood parcels reach very high numbers (19,500 ind m^−2^).

After the onset of the sulfophilic stage (sensu [16]), microbial mats and patches of black sediments developed adjacent to the whale carcass within 1.5 years; these are heavily colonized by sulfide-tolerant organisms such as dorvilleid polychaetes and by vesicomyid clams (Figure 3; [28], [32], [81]). The macrofaunal composition frequently becomes dominated by dorvilleid (>36% at all sites) and ampharetid polychaetes, the former group being composed of a multi-species complex (>40 dorvilleid spp. [32]). Macrofaunal composition around organic falls exhibits strong similarity of high-level taxa (Figure 3), with cumaceans being highly abundant at kelp- and whale-falls (>15–30%). This suggests similar community responses to organic and sulfide enrichment. The sulfophilic stage is brief at kelp falls (<0.5 y) but can last for at least 5 to 6.8 years at wood- and whale-fall sediments, with an apparent gradual re-colonization by background species.

### Cross-site multivariate comparisons

Cross-site comparisons of assembled data set revealed significant differences in macrofaunal density between chemosynthetic sites and habitats. At hydrothermal vents, vesicomyid clam beds at Middle Valley vents exhibited the highest macrofaunal density (p<0.001; Figure 2). The elevated macrofaunal densities at ampharetid bed habitats in New Zealand seeps (56,595 ind m^−2^), were significantly (or marginally significant) higher than all other sites compared here (although whale fall densities in the literature also reach these levels [e.g., [28], Table S1). Clam bed and microbial mat habitats at the shallower Californian seep sites had generally higher macrofaunal densities than microbial mat habitats at vents, frenulate fields at seeps, and organic fall habitats (F = 7,79, p<0.01; Figure 2). Clam bed habitats at Hydrate Ridge, OR and at Eel River, CA also had significantly higher densities than similar habitats at Costa Rica and the deepest Kodiak and Unimark seeps (p<0,01). Frenulate fields at San Clemente, Unimak and Kodiak seeps had similar macrofaunal densities, but these were generally lower than in other seep habitats (Figure 2).

**Figure 4:**
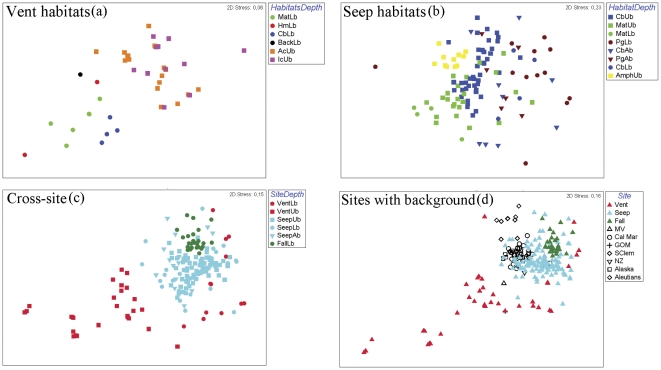
MDS plots of family-level abundance based on the Bray Curtis similarity index. Panels A–C: Squares – Upper bathyal (Ub 200–1500 m); Circles – Lower bathyal (Lb 1501–3000 m); Triangles - Abyssal (Ab>3000 m) samples. Colors indicate habitats within sites: Light green - microbial mats (Mat), Dark blue - clam beds (Cb), Red - hot muds (Hm), Orange - active venting (Ac), Pink - inactive venting (Ic), Brown - frenulate beds (Pg), Yellow - ampharetid beds (Amph); Black - Background sediments. Panel D: Symbols indicate background samples (in black) in different basins (sites).

At the family level, there is similarity between kelp-, wood-, and whale-fall infauna, which also resemble seep and lower bathyal vent sites (Figure 4-D). There is significant dissimilarity in community structure (family level) within each site, depth and habitats (Figure 4 - A–C). Vent sites from the West Pacific are remarkably different from all other seep and organic fall sites, most of which occur in the E Pacific and are thus highly separated biogeographically (ANOSIM R = 0,68, p<0.01; Figure 4 - C). Among vent sediments (Figure 4 - A), communities in the relatively shallow Manus Basin (1480 m) are significantly distinct from those at the deeper Middle Valley site (2410 m; ANOSIM R = 0,662, P<0.001). Polychaetes (syllids, dorvilleids and orbiinids) and bivalves contributed most to these differences (SIMPER, Figure 3). At Middle Valley, microbial mats were distinct from all other vent habitats (p<0.001), but clam beds and hot muds were marginally different from each other (p = 0.06; Figure 4). The dissimilarity between hot mud and other vent habitats was higher (>78%) than between microbial mats and clam beds (64%; SIMPER). Seep sites exhibited strong differences in macrofaunal community structure between upper bathyal (200–1500 m) and the other depth zones (ANOSIM R 0.603, P<0.001), but not between the two deeper zones (lower bathyal and abyssal). Differences between seep assemblages across depth zones (i.e. upper vs. lower bathyal) were especially evident between microbial mats and clam bed or ampharetid bed communities (ANOSIM R = 0,34, P<0.001). Macrofaunal communities at frenulate fields were most similar to those in clam bed sediments, but were dissimilar to those in other seep habitats (p<0.01, Figure 3). Macrofaunal communities at organic falls were not strikingly different from each other at the family level (Figures 3 and 4).

Multivariate dispersion analysis based on Jaccard's dissimilarity index indicates strong differences in macrofaunal beta-diversity among vents, seeps and organic falls (PERMDISP F = 30,8, p_perm_ = 0,001). Pair-wise comparisons indicate strong differences in beta-diversity between vent sediments and organic falls, and between vents and seeps (p<0.001). The vent fauna exhibited the widest heterogeneity among all sites (55% Jaccard's distance), whereas organic falls were more homogeneous between sites (32% on average). This homogeneity is likely a consequence of the limited biogeographic range represented by the organic fall samples.

### Species diversity

**Figure 5:**
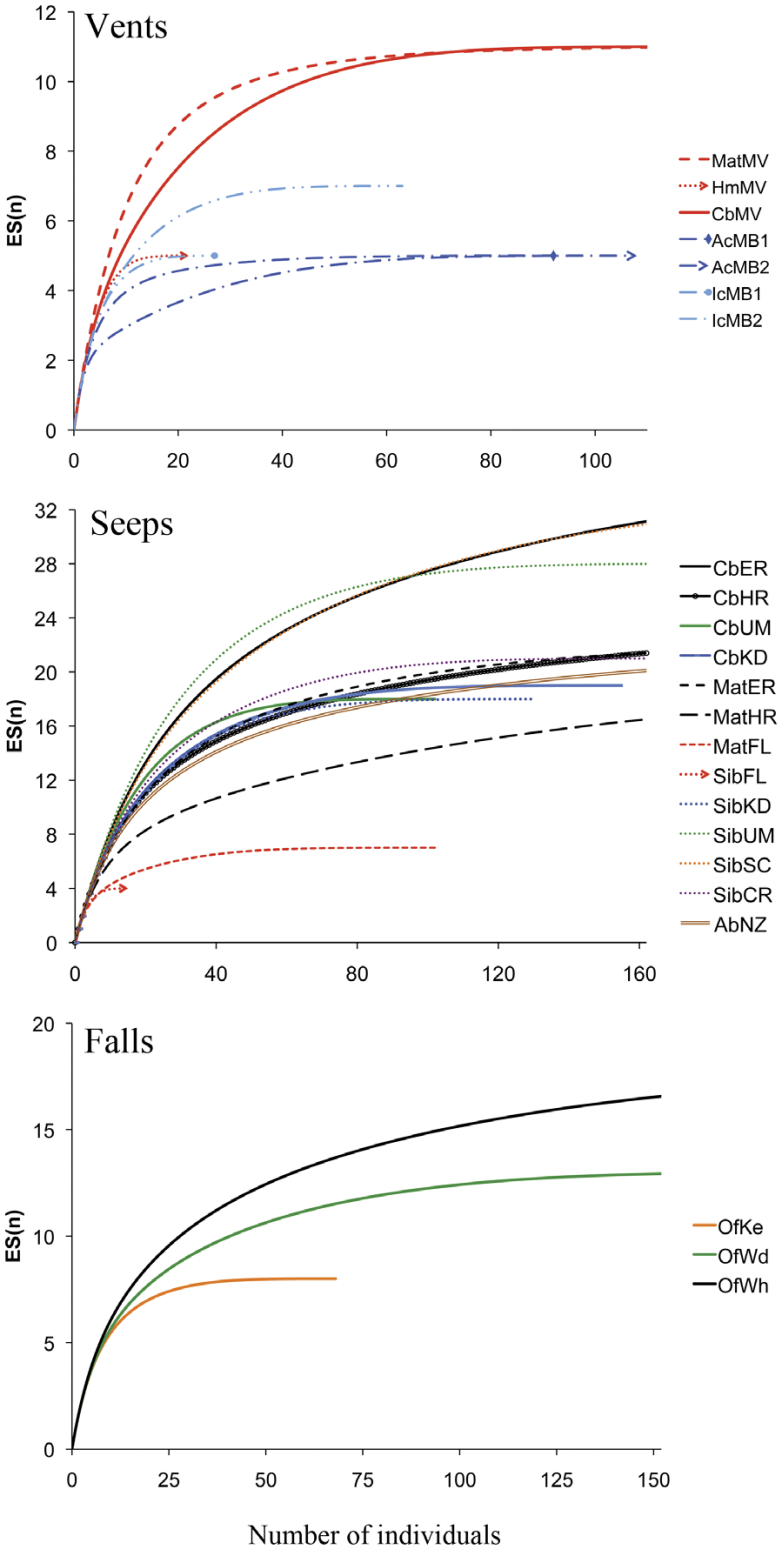
Rarefaction diversity at species level for vent (upper), seep (middle) and organic-fall (lower) habitats (cores pooled by site and habitat). Colors indicate sites; Line patterns differentiate habitats within sites. Legend: *Mat* – microbial mats, *Cb* – clam beds, *Sib* – frenulate fields, *Ab* – ampharetids beds, *Ac* – active vent sediments, *Ic* – inactive vent sediments, *Hm* – vent hot muds, Of – organic-falls. Sites: MV – Middle Valley, MB – Manus Basin, ER – Eel River, HR – Hydrate Ridge, FL – Florida Escarpment, KD – Kodiak Alaska, UM – Unimark Aleutians, SC – San Clemente Basin, NZ- New Zealand, Ke – Kelp-fall, Wd – Wood-fall, Wh – Whale-fall.

Rarefaction analysis indicated a general trend of elevated diversity at a few seep and organic-fall habitats, whereas vent sediments in general hosted lower diversity (Figure 5). At active and inactive vent sites in Manus Basin diversity was low and similar to hot mud sediments in Middle Valley (Figure 5). The highest diversity in vent sediments were found in clam bed and microbial mat habitats (Es_100_ = 11). Seep habitats exhibited a broad diversity range (Es_100_ from 4 to 27.5) compared to other sites, reflecting higher variability of geochemical conditions (Figure 5). Frenulate fields at the deeper Unimak and San Clemente seeps, together with clam beds at Eel River seeps exhibited the highest diversity of all seep habitats (Es_100_ = 27 for both habitats; Figure 5). These areas also tended to have higher evenness (J′ = 0.87 and J′ = 0.71–0.95 for clam beds at Eel River and frenulate fields at San Clemente and Unimark, respectively). A second cluster with habitats depicting “intermediate” diversity values included microbial mat habitats, organic-falls and other clam bed and frenulate fields at various sites (Figure 5). The seep habitats with lowest diversity were microbial mats at the Florida escarpment and Eel River, CA (Es_100_ = 7, and Es15 = 4, respectively) and frenulate fields of the Florida escarpment (Es_15_ = 4). However, community evenness in Florida Escarpment frenulate fields was elevated (J′ = 0.90), in contrast to the high dominance of hesionid polychaetes in microbial mats at the same site (Figure 3).

### Nutritional sources

#### Vents

Symbiont-bearing vent taxa, while very common on hard substrates, are typically limited to vesicomyid bivalves in hydrothermal sediments. Siboglinids provide an exception; *Siboglinum* spp. is present in warm sediments of Middle Valley [82] and at the Chile Triple Junction (Thurber et al. unpublished) and *Sclerolinum* sp. is present in low flow vents at Bransfield Strait, Antarctica [83]. Where studied, the vent infauna exhibits a range of nutritional sources depending on location and habitat (Table 3). At Manus Basin active sites, heavy δ^13^C signatures of most infauna (−13 to −16‰) may reflect reliance on microbes with C fixed by the reverse TCA cycle [27]. Among the reducing environments considered here, this trophic pathway appears to be unique to hydrothermal vents. At inactive sites, δ^13^C signatures (−20 to −26‰) reflect a mix of photosynthetically derived organic matter and sulfide-oxidizing microbes. In contrast, infauna in active sites in Middle Valley appear to rely largely on sulfide-oxidizing bacteria, based on lighter δ^15^N values than background fauna and average δ^13^C values of −26 to −29.5‰. Strong methane contributions to the C pool seem rare, but have been observed in the maldanid *Nicomache* sp. and *Capitella* spp. from Middle Valley and a syllid from the Chile Triple Junction (Thurber at al., unpublished). Few infaunal species within Middle Valley hydrothermal sediments appear to rely on photosynthetically derived food sources. At the Gorda Ridge, orbiniids in the clam bed (−40‰) and an aplacophoran (−37‰) in bacterial mats in hot sediments (−41.6‰), each had light δ^13^C signatures, but this may not indicate methane incorporation [84].

**Table 3:** Stable isotope signatures of sediment macrofauna from chemosynthetic sites and habitats.

System	Site	Region	Habitat	Avg δ^13^C (min/max)	Avg δ^15^N (min/max)	Ref.
Vent	Middle Valley	NE Pacific	Microbial mat	−29.5	−0.6	[27]
			active clam bed	−26.0	−1.1	
			inactive clam bed	−28.4	−0.7	
			Inactive sed	−22.1	6.6	
	Papua New Guinea	Manus Basin	active	−13/−17	7/8	[27]
			inactive	−20/−26	11/12	
Whale	Santa Cruz Basin	NE Pacific	Whale (Sulfophilic)	−36/30	−0.9/14	[16]
Kelp	Santa Cruz Basin	NE Pacific	Kelp (0 m)	(−38/−16)	(−12/12)	[17]
Wood	Santa Cruz Basin	NE Pacific	Wood (0 m)	(−30/−20)	(−2/18)	[17]
Seep	New Zealand	Builders Pencil		−22 (−30/−17)	9 (5/13)	[63]
		LM-3		−50 (62/31)	3.4 (1/8)	
		Rock Garden	Knoll	−20 (−22/−18)	10.3 (0/8)	
		Omakere Ridge	LM-9	−24 (−31/−18)	7.4 (2/12)	
			Kaka	−33 (−47/−21)	7 (−1/12)	
			Bears Paw	−45 (−54/−26)	6 (3/10)	
		Uruti Ridge		−21 (−24/−17)	10 (4/14)	
		Opouawe Bank	North Tower	−29 (−42/−20)	7 (1/12)	
			South Tower	−25 (−36/−20)	8 (1/12)	
			Takahe	−38 (−54/−21)	1 (−9/12)	
	Florida Escarpment		Black mat	−61	−3	[80]
			Microbial mat	−46	1	
			Pogo Field	−36	3	
			Clam beds	−39	1	
	Gulf of Alaska	Unimark	Pogo Field	−27 (−46/−19)	9 (−3/14)	[80]
			Clam beds	−30 (−61/−12)	9 (−1/14)	
			Non seep	−20 (−27/−11)	12 (10/15)	
		Kodiak	Pogo Field	−43 (−65/−21)	9 (2/17)	
			Clam beds	−35 (−91/−21)	7 (−1/15)	
			Non seep	−22 (−33/−18)	11 (6/14)	
	Oregon Margin	Hydrate Ridge	Microbial mat	−44	6	[68]
			Clam bed	−34	9	
			Non seep	−21	13	
	California	Eel River	Microbial mat	−22 (−36/−18)	11 (8/15)	[68]
			Clam bed	−25 (−40/−18)	10 (4/16)	
			Non seep	−21 (−31/−18)	12 (9/13)	
		San Clemente	Pogo field	−42	−1	[90]
	NW Atlantic	Blake Ridge	Clam bed	(−56/−35)	(1/11)	[110]

#### Seeps

Although methane seeps are fueled by methane, the dominant form of autotrophic production is based on the resultant sulfide apparently derived from AOM. Among all the sites studied, only a subset of the fauna obtains carbon from methane, yet the diversity of this group is surprising: ampharetids, capitellids, cnidaria, cumaceans, dorvilleids, gamarids, lumbrinerids, nereidids, maldanids, turbellarians, and phyllodocids all have isotopic signatures that indicate incorporation of methane-derived carbon ([63], [68], [80]. The extent of methane-derived carbon has been investigated at a range of seep sites. In the ampharetids beds of New Zealand seeps, the macrofauna derived 6–100% of their carbon from methane [63]. Macrofaunal tissues had up to 55% methane-derived carbon in Florida escarpment mats, 20–44% in Oregon microbial mats, Florida, OR, CA and Kodiak, AK clam beds, and Kodiak pogonophoran fields, and 9–23% in Unimark, AK clam beds and pogonophoran fields and Eel River, CA microbial mat habitats [68], [80]. Macrofauna from seep sites exhibit more variable ranges in C and N signatures than in the other systems (Table 3) and there is no strong trend in the isotopic signatures with depth or biogeographic region. Along the western Pacific continental margin, the average isotopic signature of seep macrofauna suggests stronger reliance on methane-derived carbon as depth increases [68], but even at the deepest seeps and in most habitats studied there are a substantial number of heterotrophic organisms utilizing other non-chemosynthetic food sources [80]. At local scales (i.e. between habitats within a site), the isotopic composition of methane and the methane flux rates influence the δ^13^C signatures of microbe-consuming heterotrophs.

#### Organic falls

The most abundant invertebrates colonizing whale-, kelp- and wood-fall sediments do not feed exclusively on organic carbon from the organic parcels (i.e. kelp or wood biomass). Although a high proportion (>50%) of the diet can come from the organic islands [17], [85], sediment organic carbon and bacterial carbon contribute to the diet of the opportunist species. At both kelp and wood parcels there is an input of chemosynthetic carbon via consumption of free-living bacterial mats growing on sediments and possibly over the surface of wood and kelp parcels. Dead biomass from whale falls may support chemosynthesis at early stages of decomposition (i.e. <18 months). Therefore, heterotrophic consumption of chemosynthetic and other food sources produce a broad range of macrofaunal isotopic signatures around organic falls (Table 3). Low δ^13^C signatures from organic-fall sediment macrofauna are found in cumaceans and dorvilleid polychaetes (<−35‰), but in general these signatures are within the broad spectrum of values found at vent and seep habitats. In sulphidic sediments at whale falls, infaunal biomass often appears to be dominated by vesicomyid clams [32], which rely on sulfide-based chemoautotrophic production.

### Endemicity and links to the surrounding deep sea

Hydrothermal vent sediments appear to support a mix of genera or species acknowledged to be vent/seep/whale-fall specialists (e.g., *Amphisamytha, Provanna, Depressigyra, Hyalogyrina, Paralvinella, Nereis sandersi)*, but also taxa broadly present on continental margins around the world (*Leitoscoloplos, Sphaerosyllis, Ophryotrocha*). Different sub habitats may support greater or lesser numbers of vent-endemic species [27]. Between Guaymas and Middle Valley, three infaunal heterotrophic species are shared; an ampharetid, hesionid and polynoid polychaete [14]. Among symbiont-bearers living in sediments, the tubeworms *Lamellibrachia barhami*, *Escarpia spicata*, and the clam *Archivesica gigas*, *Calyptogena packardana*, and *C. Pacifica* frequently occur at vents, seeps (e.g., [86]) and whale falls [16], [32], [87], [88]

Based on sampling of cold seep sites in the Sea of Okhotsk between 160 and 1600 m, Sahling et al. (2003) [89] concluded that seep endemic faunas were confined to depths below 370 m. While most of the symbiont-bearing invertebrates at deep-water seeps are seep- (or in some cases vent-) endemics, the degree of seep/vent endemism is significantly less among the heterotrophic infauna. The most sulphidic sediments (microbial mats dominated by *Beggiatoa* bacterium) frequently support the largest number of seep-endemic species. At the species level, Levin et al. found only about 50% of seep macroinfauna at Hydrate Ridge, OR and Eel River, CA were seep endemics, with the remainder present in nearby bathyal slope sediments. Bernardino & Smith (2010) [90] observed that about 20% of species present near tubeworm thickets were also found at nearby whale, wood, and kelp falls. Although infaunal meiofauna are not a focus here, it is notable that almost no metazoan meiofaunal genera or foraminiferal genera present at seeps are considered endemic to chemosynthetic ecosystems [70], [91], [92].

Organic-fall sediments, in particular those around whale falls, appear to host a number of endemic dorvilleid species, although many of the ∼40 species of dorvilleids collected at whale falls are still in the process of description. Some of the dorvilleid species at whale falls (including species in the genera *Ophryotrocha, Parougia and Schistomeringos*), can occur in abundance at wood falls and seeps [17], [32], [93]. Most dominant taxa present at these islands are microbial-mat grazing and predacious polychaetes as well as opportunistic cumaceans [17].

## Discussion

### Conceptual framework of reducing sediment macrofaunal diversity

**Figure 6:**
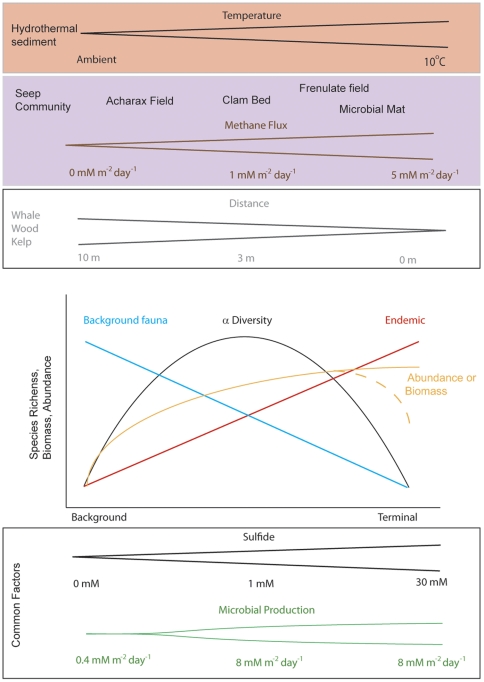
A conceptual framework of factors shaping the biodiversity, density, and biomass of macrofauna in reducing ecosystems. The top three panels highlight drivers that are unique to certain systems. The bottom two panels provide axes for features that are similar among systems (note that while values are given for these two axes the values are not consistent across the different ecosystems represented although the relative scale is). The middle panel illustrates how these factors translate into community attributes of each of the ecosystems. The bifurcation in the abundance and biomass factors indicate that, depending on the system, stress overrides high productivity in these habitats and both biomass and species richness fall bellow an intermediate level (e.g. hydrothermal sediments where the temperature stress overrides the importance of a high productivity system).

Deep-sea chemosynthetic ecosystems host a variety of geochemical and microbial processes that mediate organic carbon fixation, impose disturbance and a variety of stresses requiring physiological adaptations, and influence associations of various endemic endosymbiont-bearing species, enrichment opportunists and typical background fauna. Sediments in these chemosynthetic ecosystems usually share the presence of reduced inorganic compounds, specifically methane, hydrogen sulfide, hydrogen, or a combination of these. High flux rates of reduced chemicals (e.g. H_2_S, CH_4_) appear to be a common factor influencing the sediment-dwelling macrofauna at seeps, vents and at some organic falls (Figure 6). While these reduced chemicals in porewaters provide a cross-ecosystem similarity and have important effects on the biota, the ecosystems considered here differ in additional environmental factors that modify the local (i.e. meter scale) structure of macrobenthic communities. Therefore, seep, vent and organic-fall sediments are highly heterogeneous with respect to their geological or biological origin, their geochemical and microbial processes, and (to some extent) their evolutionary histories; they frequently exhibit a complex mosaic of habitats inhabited by a mixture of specialist and background fauna, which are in turn influenced by thermal stress (at vents), patch dynamics (seeps and organic falls) and bathymetric trends [12], [15], [16].

Our comparison of seep, vent and organic-fall sediments suggests that food availability associated with increased flux of reduced chemicals, support the highest macrofaunal densities and biomass compared to deep-sea sediments at similar depths. This observation generally supports our hypothesis that systems with elevated sulfide concentrations will exhibit enhanced density. The abundance and composition of the sediment macrofauna at vents, seeps and organic-falls is closely related to the rates of sulfide or methane production (i.e. fluid flow and geochemistry), habitat (i.e. based on dominant foundation species), and organic enrichment (at food-falls), and leads to predictable trends in macrofaunal communities relative to background assemblages (Figure 6). Increasing levels of stress or disturbance selectively exclude non-tolerant background macrofauna from most chemosynthetic habitats at seeps, vents and organic falls. As the levels of sulfide, methane or organic enrichment increase within the sediments, macroinfaunal abundances typically increase. In sediments nearby organic falls, macrofaunal abundances are consistently higher in sediments with high organic enrichment [16], [17], which is in accordance with the Pearson and Rosenberg SAB model [94]. High macrofaunal densities at seep habitats (e.g. clam beds, microbial mats and ampharetids beds) are associated with increased dominance of polychaetes; this is a common pattern in various seep habitats at the Gulf of Guinea, Nile Delta, Gulf of Mexico and the Mediterranean [25], [95]. However, macrofaunal abundance in sulfide-rich sediments at seeps and vents may be lower than background sediments as a result of increased sulfide flux rates, decreased sediment stability, and/or high temperatures [63].

Macrofaunal composition and diversity at seeps, vents and organic falls are tightly associated with the sediment geochemistry (e.g. levels of sulfide or organic content; Figure 6). Many of the common families and genera of symbiont-bearing and heterotrophic taxa characteristic of these systems are encountered in highly sulphidic sediments. The seep infauna is a mixture of background, sulfide-tolerant and endemic species, and most sulphidic habitats are dominated by polychaetes (Dorvilleidae, Hesionidae, Ampharetidae), gastropods and peracarid crustaceans. Capitellid polychaetes also appear to tolerate these inhospitable sediments, yet the diversity within the seep Capitellidae is poorly constrained and requires further molecular analysis. Survival strategies for some species living deep in seep sediments remain enigmatic, including for a newly discovered spionid polychaete living >10 cm down in the sediments in anoxic and highly sulphidic sediments off New Zealand [17]. Dorvilleid polychaetes are extremely abundant and diverse at organic falls in the NE Pacific [61], [93], [96]; there are approximately 14 and 40 species of dorvilleids in seep and whale-fall sediments, respectively [62], [97]. Whereas a diversity of dorvilleid and capitellid polychaetes appear to have a physiological ability to withstand high sulfide settings, other species either oxygenate the sediment to reduce chemical stress or are restricted to the oxygenated portion of the sediment column. Clams and siboglinid polychaetes bioirrigate the sediment, increasing the vertical penetration of oxygen [19]. Ampharetid polychaetes appear to use an alternate approach holding their brachia out of the seep sediment while inhabiting vertical tubes, ameliorating sulfide stress [31]. Such adaptations may also occur at organic-rich whale fall sediments inhabited by the ampharetid *Glyphanostomum* sp. nov. [43]. A diversity of fauna occur at the sediment surface in reducing habitats, including many gastropods and hydroids, which can be numerically dominant (Table S1; Figure 3). Syllids (including *Sphaerosyllis* sp. as in the hydrothermal sediments) and hesionids also are frequently abundant taxa in the most sulphidic sediments; and cumaceans, amphipods, and isopods can also be abundant in certain locations. Vent sediments with high temperatures harbor a very distinct macrofauna relative to seeps and organic falls being dominated in some instances by spionid polychaetes (genus *Prionospio* (*Minuspio*)), syllids and orbiinid polychates. Nuculanid bivalves (*Nuculana* spp.) are a widespread group common in Manus Basin active and inactive sediments, and in Guaymas Basin [27].

Diversity is highly variable in many seep habitats and generally lowest at high-temperature sediments of hydrothermal vents, which is consistent with our hypothesis that systems with high sulphidic concentrations and/or high temperatures will have reduced diversity. Hydrothermal vent sediments communities are less diverse than all other chemosynthetic ecosystems (Figure 5), suggesting that temperature stress may limit macrofaunal colonization. This is supported by higher diversity in microbial mat and clam bed sediments of vents relative to active (hot) and inactive sediments (Figure 5; [25], [97], [98], [99]). Diversity was generally higher in seep habitats with lower macrofaunal dominance that are apparently less sulphidic; but this pattern was not universal. For example, siboglinid beds on the Alaska margin were highly diverse but not off Florida. The same pattern is found in microbial mats along the California margin (ER and OR). The heterogeneous geochemical conditions at seep habitats at scales of meters may cause substantial heterogeneity in local diversity in habitats that appear similar visually [17].

### Taxonomic and trophic similarities

**Figure 7:**
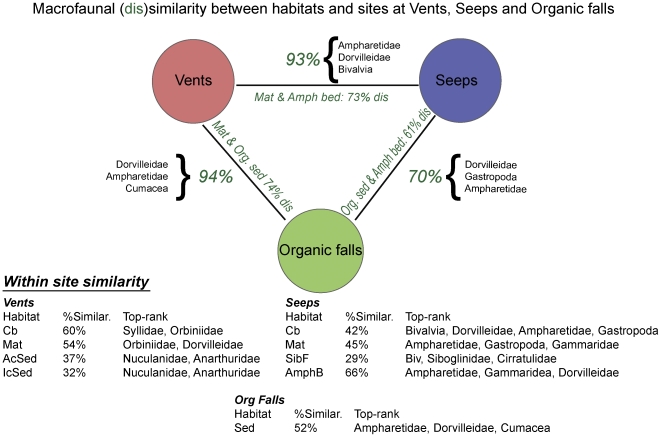
Diagram showing degree of community similarity or dissimilarity between chemosynthetic ecosystems and habitats. Values outside bars denote average dissimilarity between sites with all habitats combined and taxa responsible for those differences. Values inside bars indicate the lowest dissimilarity between two habitats among the two sites compared. *Legend:* Green color – indicates dissimilarity percentages from SIMPER analysis.

Multidimensional analyses reveal that vent, seep and organic fall macrofauna are distinct (Figure 7). The highest community similarity was observed among kelp, wood and whale falls, which share many dominant macrofaunal taxa (e.g., dorvilleid and ampharetids polychaetes, cumacean species) where sulfide concentrations are high [27], [100]; but this similarity may be explained in part by the small biogeographic range represented in our data set (NE Pacific). Vent sediments host different macrofaunal communities than seeps and organic falls. The vent macrofauna responds to local-scale (i.e. meter) processes linked to the habitat types, but also reflects regional-scale isolation between the Western Pacific and NE Pacific provinces [16], [100]. This species-level segregation of the vent macrofauna is in marked contrast to the high generic overlap of dominant chemosymbiotic megafauna that is found in seeps, vents and whale falls [15], [25], [101]. The seep macrofauna do not show a systematic response in terms of species composition to habitat heterogeneity at local and regional scales, and to depth trends [102]. There is a clear separation of clam bed and microbial mat-associated macrofauna between lower and upper bathyal sites (Figure 4), but this was not true for the frenulate field macrofauna. The New Zealand ampharetid beds appear to host a distinct upper bathyal fauna and may characterize a new habitat type for seep settings [100].

Our study supports distinctions of vent, seep and organic-fall macrofaunal assemblages at broad scales (ß diversity). The vent fauna exhibited the widest heterogeneity among all sites supporting distinct evolutionary origins [15], [16], [103]. While sharing some species, organic falls and seeps may clearly harbor distinct faunas at bathyal and abyssal depths (e.g., [86]). Therefore, although high sulphidic sediments usually lead to macrofaunal communities dominated by a few heterotrophic and symbiont-bearing species at seeps, vents and organic falls, there appears to be limited overlap between these ecosystems at the species level (Figure 7). Megafauna in contrast, share 20% of species at vents and seeps in close proximity off Japan [104].

Most macrofaunal species (and hence most of the species richness) in vent, seep and organic-fall sediments are heterotrophic, with a limited number hosting chemoautotrophic symbionts (e.g. siboglinid polychaetes, and vesicomyid and solenid bivalves). The chemosynthetic production available to the heterotrophic fauna is mostly derived from free-living chemoautotrophic microbes rather than sinking detrital organic matter ([63], [68], [80]; Table 3). The biogeochemical differences between vent, seep and organic-fall sediments result in wide differences in organic utilization (i.e., food sources) and in the degree of chemoautotrophic nutrition. Macrofauna from seeps probably exhibit the widest range in isotope signatures due to the input of isotopically light methane, which provides an addition source of microbial production available to heterotrophic fauna [15], [27], [60], [63]. The most depleted δ^13^C signatures at seeps come from microbial-mat habitats and from New Zealand ampharetid beds, which are likely a result of high rates of archaeal methane oxidation and/or sulfide flux [31]. The use of methane by macrofauna is not well documented at whale falls, even though methane concentrations can reach 2.9 mM at whale falls [105]. Vents can also sustain methane input, and methane-derived carbon has been detected in some vent macrofauna (mentioned above), but hydrothermal methane does not have a unique isotopic signature [15] making it difficult to identify methanotrophy in vent habitats. In all of the reducing ecosystems, a broad range of macrofaunal isotope signatures indicates that the input of chemosynthetic carbon is inconstant in time or locally and that there is additional input of photosynthetic food sources. There is evidence for higher input of photosynthetic carbon to shallower seep sites at the California margin [99], [106]. Not surprisingly, macrofauna from organic falls exhibit a broad range of isotope signatures consistent with a variety of food resources at these islands, with the dominant dorvilleid polychaetes and cumaceans exhibiting higher degrees of chemoautotrophically based nutrition.

### Depth trends and zonation

It is still unclear if the macrofauna exhibit depth zonation across chemosynthetic sediments in the deep sea. Strong depth zonation of seep megafauna has been documented in the Gulf of Mexico [89], and in the Sea of Okhotsk [107], but comparable studies have not been done for most infauna. A major exception is for the family Vesicomyidae, which occurs at depths from 100 to 9,000 m but with strong depth zonation for most genera [107]. Nine genera were restricted to a single bathymetric zone, seven had bathyal distributions and two were abyssal [86]. Several families of other taxa that have radiated in chemosynthetic sediments (Ampharetidae, Dorvilleidae, Hesionidae, Polynoidae) are now subject to molecular evolutionary studies. Some species found at both vents and seeps in close proximity have been shown to share haplotypes across these ecosystems. In some cases, there are affinities (at species level) with shallow water representatives (e.g., Dorvilleidae [108]). A key question remaining to be addressed involves the relative importance of connectivity (e.g., geographic isolation) versus habitat geochemistry in determining the faunal similarities across the different reducing environments considered here.

### Concluding remarks

Deep-sea chemosynthetic sediments provide a mosaic of habitats that offer an evolutionary opportunity to adapt to extreme, energy-rich environmental conditions that have excluded much of the background deep-sea fauna. Although the macrofaunal structure (family level) of vent, seep and organic falls exhibit some commonalities such as low diversity and high dominance of a few polychaete taxa, community-level analyses reveal strong differences in community composition between these ecosystems. These differences are likely to result from different regimes of physiological stress (e.g., high temperatures, high sulfides, low oxygen), from population and community-level processes including predation and facilitation, and from poorly known depth trends, biogeographic isolation and evolutionary divergence. Broad-scale analysis suggest that macrofaunal assemblages in chemosynthetic sediments exhibit a low degree of similarity at the species level across systems, making them more susceptible to increasing human extractive and disposal activities (reviewed in [27]).

## Supporting Information

Table S1 **Summary of comparable work on macrobenthos community structure in bathyal hydrothermal vent sediments, cold seeps, whale-, wood- and kelp-falls.** This table includes additional sites not cited in the text [111], [112], [113].
(DOCX)
