# Supplementary material for: Comparative Composition, Diversity and Trophic Ecology of Sediment Macrofauna at Vents, Seeps and Organic Falls
Source: PLoS One. 2012 Apr 4;7(4):e33515. doi: 10.1371/journal.pone.0033515 (PMC3319539; doi:10.1371/journal.pone.0033515)
Supplement: Table S1 — Summary of comparable work on macrobenthos community structure in bathyal hydrothermal vent sediments, cold seeps, whale-, wood- and kelp-falls. This table includes additional sites not cited in the text [111], [112], [113]. (DOCX) [file pone.0033515.s001.docx]

| **Site** | **Habitat** | **Region** | **Depht (m)** | **No. macrofauna/m^2^** | **Rank 1 dominance (Density)** | **Dominant taxa** | **Other taxa** | **Diversity patterns** | **Ref.** |
| --- | --- | --- | --- | --- | --- | --- | --- | --- | --- |
| **Escanaba Trough** | Hydrothermal sediment + near background | NE Pacific | 3254 | 18,709 |  | Ampharetidae, Orbiniidae, Spionidae | nuculanid, *Provanna, Neolepetopsis* |  | [42] |
|  | Near wood |  |  | 16,932 |  | Dorvilleidae, Hesionidae, *Provanna* |  |  |  |
|  | Hydrothermal mounds |  | 3274 |  | <8% | Cirratulidae, Spionidae, Paraonidae | similar to ambient |  |  |
| **Gorda Ridge** |  | NE Pacific | 3200 |  |  |  | *C. phaseoliformis* | No species in common with non-vent sediments | [43] |
| **Guaymas Basin** | Petroleum sediment | Gulf of CA |  | 1,200-5,443 |  | Hesionidae, Dorvileidae |  |  | [43] |
|  | Clam bed |  |  |  |  | nuculanid bivalves |  |  |  |
|  | Microbial mat |  |  |  |  | *Amphisamytha* (Ampharetidae) |  |  |  |
|  | Background |  | 3270 | 1,200-1,900 |  |  |  |  |  |
| **Galapagos Mounds** | Sulphidic sediments | N Pacific | 2700 |  |  | *Aurospio* (Spionidae) |  | Reduced species richness and density relative to non vent. | [18] |
| **21o N, EPR** | Hydrothermal sediments |  | 2717 |  |  | *Anobothrus* sp. (Ampharetidae) Thyasiridae |  | Low diversity | [18] |
| **Middle Valley, Juan de Fuca** | Microbial mat | NE Pacific | 2406-2414 | 5,589+2022 | 14% | *Leitoscoloplos pachybrachiatus*, Dorvilleidae spp., Ampharetidae spp. |  |  | [27] |
|  | Hot mud |  |  | 910±263 | 50% | Syllidae, Spionidae | Dorvilleidae, Gastropoda |  |  |
|  | Clam bed |  |  | 16,704±2247 | 34% | Orbinidae, Syllidae | Exogoninae |  |  |
|  | Background |  |  | 3,120 |  | Tanaidacea | *Prionospio* sp. |  |  |
| **Solwara 1, Manus Basin** | Active mud | Papua New Guinea | 1511-1575 | 934 | 36% | *Paraleptognathia* (Tanaidacea) | Isopoda, Nuculanid bivalve, *Heteromastus* |  | [27] |
|  | Inactive mud |  | 1504-1634 | 445 | 58% | *Paraleptognathia* (Tanaidacea) | nuculanid bivalve |  |  |
| **South Su, Manus Basin** | Active mud | Papua New Guinea | 1312-1369 | 3,740 | 77% | *Prionospio (Minuspio)* | nuculanid bivalve | Reduced diversity relative to inactive sediments | [27] |
|  | Inactive mud |  | 1356-1452 | 908 | 43% | nuculanoid bivalve | *Paraleptognathia* sp., *Prionospio* sp. |  |  |
| **Whale, Wood and Kelp falls** | | | | | | | | | |
| NE Pacific  Whale | Disturbed or organic rich sediments (0.12 to 6.8 yrs) | Santa Cruz Basin  (30 ton carcass) | 1670 | 1,820 to 51,455 | 41-86% | Dorvilleids  Amphipods  Cumaceans  Gastropods  Bivalves | Ampharetids | Higher diversity at peak of sulfides | [28,29] |
|  | Clam shell sediments (0-0.5m) not organically rich | Santa Catalina Basin (> 4yr – 36yr carcass) | 1240 | 3,333 | 32-37% | *Levinsenia oculata* |  | Bkgd species dominant;  Higher diversity at 0m | [13] |
|  | Sediments 4 months after carcass placement | San Diego Trough (5 ton carcass) | 1220 | 20,000 to 45,000 |  | *Ophryotrocha*  Chrysopetalid polychaetes | *Cumella* sp. |  | [28] |
|  | Sediments (0-0.5m) | Monterey Bay (2-3 yr carcass) | 2893 | 6,756 to 40,537 | 40-49% | Dorvilleids  Ampharetids  Spionids |  | High density and low diversity | C.R Smith unpublished |
| NE Pacific  Wood | Organic rich sediments (5.5 yrs, 0m) | Santa Cruz Basin | 1670 | 15,079±2,359 | 29% | *Ophryotrocha*  Ampharetidae | Dorvilleids (*Parougia, Pseudophryotrocha*) | Reduced diversity at peak of sulfides (> 3 yrs) | [17] |
| NE Pacific  Kelp | Sediments (3mo, 0m) | Santa Cruz Basin | 1670 | 5,286±997 | 36% | *Ophryotrocha*  *Cumella*  Ampharetidae | *Hyalogyrina* (Gastropod) |  | [17] |
| **Seeps** | | | | | | | | | |
| Gulf of Alaska | Frenulate Field | Kodiak | 4413-35 | 6,622 | 31% | Bivalve (unid.) | Amphartidae | Highest richness and diversity off-seep, lowest richness in clam bed but highest evenness (j') | [80] |
|  | Clam bed |  | 4414-44 | 3,426±322 | 14% | Amphareidae | Cirratullidae |  |  |
|  | Background |  | 3427-480 | 3,749 | 20% | Spionidae | Tanaidacea, Cirratulidae |  |  |
| Aleutians | Clam bed | Unimak | 3267 | 2,839±889 | 19% | Tanaidacea | Ampharetidae, tainadacea | Frenulate field highest richness but background highest diversity and microbial matt highest evenness (j') | [80] |
|  | Frenulate Field |  | 3283 | 9,739±2,342 | 17% | Gastropoda | Nemertea, Cirratulidae |  |  |
|  | Reference |  | 3165-310 | 5,362±185 | 17% | Gammarid Amphipod | Tanaidacea, Cirratulidae. Cossuridae, Paraonidae |  |  |
| Oregon Margin | Microbial mat |  | 770 | 8,869±2,762 | 48% | Gastropod | Ampharetidae, dorvilleidae |  | [19,20] |
|  | Clam bed |  | 770 | 4,968±1,620 | 23% | Tubificidae | Dorvilleidae, Ampharetidae, Gastropoda |  |  |
|  | *Acharax* bed |  | 770 | 1,310±120 | 26% | Orbiniidae | *Acharax*, Opheliidae, Cirratulidae, maldanidae |  |  |
|  | Reference |  | 770 | 327±75 | 36% | Paraonidae |  |  |  |
| California Margin | Microbial mat |  |  | 20,730±5,970 | 72% | Dorvilleid | Gammaridae |  | [20,61,109] |
|  | Clam bed |  |  | 30,424±5,181 | 32% | Dorvilleid | Gastropoda, Gammaridea, Tanaidacea |  |  |
|  | Reference |  |  | 12,836±816 |  |  |  |  |  |
| Southern California Borderland | Frenulate Field |  |  | 7,071±1,362 | 21% | Tanaid | *Sphaerosyllis*, Thyasiridae |  | [90] |
|  | Reference |  |  | 4,160 | 10% | *Prionospio* | *Aurospio*, *Exogone*, cumacea |  |  |
| Costa Rica | Clam bed |  |  | 8,024±1,547 | 16% | Hesionidae | Dorvilleidae, Cirratullidae, Spionidae |  | Levin and Mendoza, unpubl. |
|  | Microbial mat |  |  | 21,382±4,260 | 52% | Hydroids | Dorvilleidae, Cirratullidae, Ampharetidae, Lacydonidae |  |  |
|  | Reference |  |  | 7,464 |  |  |  |  |  |
| New Zealand | Ampharetid bed |  |  | 56,595±4,999 | 44% | Ampharetidae | Dorvillidae, Gammaridea, cumacea |  | [71] |
|  | Reference |  |  | 4,669 | 25% | Oweniidae |  |  |  |
| Gulf of Mexico | Microbial mats | Florida escarpment | 3300 | 20,961±11,618 | 79% | *Orseis* sp. |  |  | [80] |
|  | Frenulate fields | Florida escarpment | 3300 | 926±132 | 28% | Dorvilleid |  |  |  |
|  | Background |  | 3300 | 264±152 | 67% | Gammarid amphipod |  |  |  |
| Gulf of Mexico | Microbial mats | Green Canyon | 700 | 61,200 | 17% | *Ophryotrocha* sp. | Kefersteinia (Hesionidae); *Glyphanostomum* (ampharetidae); Chaetozone (Cirratiulid) |  | [111] |
|  | Microbial mats | Atwater Canyon | 1934 | 156,750 | 35% | *Ophryotrocha* sp. | Syllidae; Cirratulidae; Capitellidae (mediomastus) |  |  |
|  | Microbial mats | Blake Ridge Diapir | 2155 | 600±600 | 76% | *Ophryotrocha* sp. | Hesionidae; Cirratulidae |  |  |
|  | Mussel beds | Blake Ridge Diapir | 2155 | 4,750 | 50% | Bivalve (unid.) | Hesionidae |  |  |
|  | Reference | Blake Ridge Diapir | 2155 | 1,050 | 100% | Gastropod |  |  |  |
|  | Mussel beds | Green Canyon | 650 | 688 | 42-72% | *Methanoaricia dendrobranchiata* | *Bathynerita naticoidea* (Mollusca, Neritidae); | 2mm mesh | [112] |
| Nile Delta | Microbial mats | Amon Mud Volcano | 1150 | 1,600±912 | 33% | Dorvilleid | Capitellidae |  | [69] |
|  | Reference | Amon Mud Volcano |  | 766±281 | 29% | Spionidae |  |  |  |
|  | Microbial mats | Pockmark | 1700 | 2,783±782 | 50% | Dorvilleidae |  |  |  |
|  | Microbial mats | Cheops Mud Volcano | 3000 | 4,150 | 54% | Spionidae | Hesionidae |  |  |
| Marmara Sea | Bioturbated sediment |  | 1122 | 9,300 | 20% | Paraonidae | Paraonidae; spionidae; Lucinidae |  | [95] |
|  | Reduced sediment |  |  | 3,433 | 6% | Dorvilleidae | Ampharetidae |  |  |
| Gulf of Guinea | Siboglinid tube worms |  | 3160 | 6,583 | 28% | Ampharetidae | Syllidae | Highest diversity in background | [25] |
|  | Mussel bed |  |  | 22,306 | 38% | Capitellidae | Cossuridae, Playhelmenthes, Spionidae |  |  |
|  | Clam bed |  |  | 4,209 | 24% | Nemertea | Pilargidae, Capitellidae |  |  |
|  | Seep edge |  |  | 2,811 | 13% | Tanaids | Cirratulidae, Isopoda, Bivalvia |  |  |
|  | Reference |  |  | 2,447±309 | 15% | Spionidae | Bivalve, Tanaidacea |  |  |
| Håkon Mosby | Grey microbial mat | Norwegian margin | 1256 | 1,000 |  | Gastropod | Tanaid, Unidentified polychaete |  | [113] |
|  | White microbial mat |  | 1258 | 1,352±909 |  | Capitellid polychaetes | Questidae and Dorvilleidae | Lowest diversity of region |  |
|  | Frenulate field – *Oligobrachia haakonmosbiensis* |  | 1264 | 67,741±14743 |  | Dorvilleidae | Capitellidae | Second highest diversity of region |  |
|  | Frenulate field – *Sclerolinum contornum* |  | 1264 | 6,389±1,888 |  | Ampharetidae | Amhipods, Questidae, Paraonidae | Greatest taxonomic richness of region |  |
| Nyegga | Frenulate field – *Sclerolinum contornum* |  | 732 | 5695±1,583 |  | Paraonidae | Tanaids, Lumbrineridae, Amphinomidae |  | [113] |
|  | Pingo – *Sclerolinum contornum* |  | 732 | 42,694 |  | Gastropod | Capitellidae, Dorvilleidae |  |  |
| Storegga | Frenulate field – *Sclerolinum contornum* |  | 732 | 11,944±5,444 |  | Lumbrineridae | Bivalves, Amphinomidae, Questidae |  | [113] |
